# Supplementary material for: BrlR from Pseudomonas aeruginosa is a receptor for both cyclic di-GMP and pyocyanin
Source: Nat Commun. 2018 Jul 2;9:2563. doi: 10.1038/s41467-018-05004-y (PMC6028453; doi:10.1038/s41467-018-05004-y)
Supplement: Supplementary file 1 — Supplementary Information [file 41467_2018_5004_MOESM1_ESM.pdf]

Supplementary information

BrlR from *Pseudomonas aeruginosa* is a receptor for both cyclic di-GMP and pyocyanin

Wang et al.

Supplementary Note 1

BrlR–c-di-GMPs interactions in two separate c-di-GMP binding sites

In the first binding site, c-di-GMP is half accommodated by the antiparallel β-sheet and two residues (M1 and D35). In detail, the backbone amide protons of the first M1 residue form a hydrogen bond (H-bond) with the phosphate oxygen atom of c-di-GMP, and the side chain of R31 forms two H-bonds with the Gua1 base. The residue D35 also forms an H-bond with the Gua1 base. The aromatic ring of Y40 interacts with the Gua1 base through a hydrophobic π-π interaction. In addition, the hydroxyl group of the Y270 side chain from another subunit interacts with the phosphate 2 of c-di-GMP via an H-bond (Fig. 2d). In the second binding site, half of the c-di-GMP ligand is located on helix α4 of BrlR. The c-di-GMP forms only two H-bonds with the side chains of R67 and R86 via the same Gua1 oxygen atoms (Fig. 2e), thus indicating that the second site should have a lower binding affinity. The c-di-GMPs are also stabilized by strong base stacking interactions between mutually intercalated guanine groups in both the c-di-GMP binding sites (Fig. 2d,e).

Supplementary Note 2

The FP binding assays of c-di-GMP and BrlR variants

To evaluate the interactions between BrlR and c-di-GMP, all residues in two separate c-di-GMP binding sites that interact with c-di-GMP were mutated to alanine respectively, and the effects of these mutations on c-di-GMP binding were tested. The multiple-residue mutants of BrlR (R31A/D35A/Y40A/Y270A named C2E mut1) containing a mutant first c-di-GMP binding site and a wild-type second c-di-GMP binding site is defective in c-di-GMP binding, revealing a K_D of 36.2 ± 2.5 μM (a five-fold reduction relative to that of wild-type BrlR). The other BrlR mutant (R67A/R86A named C2E mut2) containing a wild-type first c-di-GMP binding site and a mutant second c-di-GMP binding site is also defective in c-di-GMP binding, revealing a K_D of 21.6 ± 1.6 μM (a three-fold reduction relative to that of wild-type BrlR) (Fig. 2g). For c-di-AMP, the C2E mut1 is also defective in c-di-AMP binding with a K_D of 63.7 ± 5.1 μM, while the C2E mut2 still binds c-di-AMP efficiently with a $\mathrm{K_D}$ of 10.3 ± 0.2 μM (Figure 2i). These results confirm that both c-di-GMP binding sites of BrlR contribute to c-di-GMP binding and that c-di-AMP only binds to the first c-di-GMP binding site.

Both c-di-GMP binding sites are in the DNA-binding domain of BrlR. However, a sequence alignment analysis showed that these residues in contact with c-di-GMPs do not belong to the conserved DNA-binding sequence of the MerR family proteins (Supplementary Fig. 5a). To further investigate whether each single amino acid of two separate c-di-GMP binding sites is equally important for c-di-GMP binding, BrlR mutants R31A, D35A, Y40A, R67A, R86A, and Y270A were prepared for the FP assays (Supplementary Fig. 6a). The results show that R31A (K_D = 42.7 ± 5.4 μM), Y40A (K_D = 34.2 ± 2.6 μM), and Y270A (K_D = 40.8 ± 3.8 μM) have a binding affinity similar to that of C2E mut1 (K_D = 36.2 ± 2.5 μM, Fig. 2g), whereas D35A (K_D = 17.5 ± 0.9 μM) shows little effect on the binding affinity of C2E mut1, indicating that residues R31, Y40, and Y270 play a decisive role in the first c-di-GMP binding site. The binding affinity of R67A (K_D = 16.4 ± 1.0 μM) is higher than that of C2E mut2 (K_D = 21.6 ± 1.6 μM, Fig. 2g), and the R86A mutant has a K_D value like that of C2E mut2, indicating that R86 is more important than R67 in binding the second c-di-GMP. Circular-dichroism (CD) spectroscopy assays show that these site-directed mutations do not cause major changes in the structure of BrlR (Supplementary Fig. 6b).

Supplementary Note 3
The effects of c-di-GMP on the DNA-binding of BrlR variants

The amount of wild-type BrlR-DNA complex formed in the presence of c-di-GMP is 5.5 times as much as that in the absence of c-di-GMP. C2E mut1 and C2E mut2 however, only showed a 1.5-fold and a 1.9-fold increase, respectively. The EMSA results of single-point mutations showed that mutations of R31A, Y40A, and R86A dramatically decrease the DNA-binding ability of BrlR. D35A and R67A slightly decrease the DNA-binding ability of BrlR (Fig. 4d,e). Consistent with the aforementioned c-di-GMP binding analyses (Supplementary Fig. 6a), c-di-GMP enhances the DNA binding of all BrlR variants with a single mutation. We know that WT-BrlR binds ~5.5-fold more DNA in the presence of c-di-GMP than in the absence c-di-GMP. For R31A, Y40A, Y270A, and R86A, the fold numbers are ~1.3, ~1.1, ~3.4, and ~1.5, respectively, while the fold numbers for D35A and R67A are ~4.8 and ~5.2, respectively (Fig. 4d,e)

The β-galactosidase activity assays showed that C2E mut1 had lower activity than C2E mut2 (115 U *vs* 167 U). C2E mut3 showed nearly the same activity as the background (Fig. 4g). These results indicated that both c-di-GMP binding sites are involved in modulating the binding of BrlR to its own promoter. Under the current test conditions, wild-type BrlR showed a 3.3-fold increase in DNA binding at high c-di-GMP concentration versus that at low c-di-GMP concentration, while C2E mut1 and C2E mut2 showed a 2.0-fold and a 2.2-fold increase, respectively. The β-galactosidase activity of C2E mut3 showed no response to the increase in c-di-GMP concentration (Fig. 4g).

Supplementary Note 4
Conformational changes of two separate c-di-GMP binding sites induced by c-di-GMP binding

In the first c-di-GMP binding site, the bound c-di-GMPs cause the ‘wing’ loop of the DNA-binding domain to rotate through interactions with R31 and D35. The twisted side chain of R31 makes a bend in β1 strand. The H-bond between c-di-GMP and Strand β1 stabilizes this strand and makes it extend lightly. In addition, c-di-GMP twists the side chain of Y40 significantly via a π-π interaction, which pushes Strand β2 close to Strand β1 and makes a bend in the subsequent helix α3. The M1 residue is relocated at the interaction between its main chain and c-di-GMP, pulling the backbone of the first loop, thus resulting in significant movement in the next helix α1. The movement of helix α1 alters the interface of the two terminal domains in the BrlR homotetramer, transforming helix α6’ and narrowing the width of the groove on the BrlR-C domain. Hence, the conformational changes in the BrlR-C domain are driven by the HTH motif and the central coiled-coil helix, thus producing a driving force in the presence of c-di-GMP (Fig. 3c). The second c-di-GMP makes the side chains of R67 and R86 twist because of the interactions between each residue and the same oxygen atom of c-di-GMP. The interactions narrow the distance between R67 and R86 and decrease the angle between helix α4 and helix α5, each of which contains an arginine residue. The twisted R67 and R86 also screw the turn of each helix (Fig. 3d).

Supplementary Note 5
The multidrug-binding domain of BrlR binds diverse toxic compounds

We demonstrated that BrlR binds the universal second messenger c-di-GMP via its DNA-binding domain. BrlR is a member of multidrug transport activators in the MerR family that can adopt a variety

of multidrug inducers in their C-terminal multidrug-binding domains[1-3]. Structural analysis indicated that the BrlR-C domain is conserved among the MerR family members (Supplementary Fig. 3b). The domain's structure is most similar to the structure of SAV2435 (PDB code: 5KAV), with a Z-score of 17.2 and an r.m.s. deviation of 2.8 Å for 142 equivalent Cα positions in a *DALI* search[4]. Despite the lack of overall sequence conservation (Supplementary Fig. 5b), residues that interact with RH6G in SAV2435 are present in BrlR. An imidazole molecule is present in the putative RH6G binding pocket of the BrlR–c-di-GMP structure, and it interacts with the aromatic ring of W150 through a π-π interaction (Fig. 5a), thus indicating that BrlR may likewise have a ligand-binding site in this canonical structural motif. Because SAV2435 binds ethidium bromide (EB) as well as RH6G[1], and the structures of EB and RH6G are similar (Fig. 5b), we hypothesized that BrlR might also bind EB. Indeed, the BrlR-C domain in native gel was readily stained by EB, but BrlR mutant W150A had a much weaker binding affinity for EB. Other mutants affecting the putative ligand binding pocket also exhibited weaker EB binding at the same protein concentration (Fig. 5d).

To test whether the BrlR-C domain recognizes antibiotics, SPR experiments were performed with a variety of antibiotics. BrlR was first immobilized on an SPR sensor chip and increasing concentrations of different antibiotics were then applied to the immobilized BrlR. The results indicated that tobramycin binds to BrlR with a low binding affinity (K_D = ~0.64 mM) (Supplementary Fig. 12a). Gentamicin also binds BrlR with a K_D of ~0.47 mM (Supplementary Fig. 12b). Responses to norfloxacin and tetracycline were also observed, but the signals were not sufficiently high to allow for the calculation of the K_D values (Supplementary Fig. 12c,d).

Moreover, it was observed that BrlR binds the fluorescein with a K_D of 33.6 ± 1.6 μM (Fig. 5c), therefore, we sought to confirm that BrlR preferentially binds c-di-GMP instead of the fluorophore in FP binding assays. The assay was performed using BrlR-C that cannot bind c-di-GMP, and results showed that BrlR-C binds F-c-di-GMP with a K_D of 88.5 ± 9.9 μM (Supplementary Fig. 11a). This K_D value is ten-fold higher than that of BrlR, indicating that the K_D value obtained using F-c-di-GMP (Fig. 2g) is mainly contributed by the interaction between BrlR and c-di-GMP. Notably, the F-c-di-GMP binding to BrlR-C can be outcompeted by EB or tobramycin but not by c-di-GMP (Supplementary Fig. 11b), which suggests that the fluorophore of F-c-di-GMP binds to the same site on BrlR-C as EB and tobramycin.

Supplementary Note 6

More dimers than tetramers formed after cross-linking of BrlR

We have found that BrlR is a tetramer by the gel-filtration as well as the analytical ultracentrifugation assays (Fig. 1f and Supplementary Fig. 1). We did observe trace amount of tetramer after cross-linking. However, much more dimers than tetramers were detected (Supplementary Fig. 13a). The native gel analysis of the same reactions showed that cross-linked BrlR is still a single band like the wild type protein, indicating that cross-linked BrlR is still a homogeneous oligomer. The faster migration rate of the modified BrlR most probably results from the reduction of positive charges due to the modification of the amines (Supplementary Fig. 13b). We then performed gel filtration assay to verify that most of the cross-linked BrlR is still a tetramer in solution (Supplementary Fig. 13c). The slightly bigger elution volume of the modified BrlR may result from its slightly bigger molecular weight and volume. The inconsistence of the results from different experiments may result from the chemical nature of

cross-linking reaction between protein BrlR and the dithiobis (succinimidyl propionate)-DSP cross-linker. DSP has an amine-reactive N-hydroxysuccinimide (NHS) ester at each end of an 8-carbon spacer arm. The spacer length of DSP is 12.0 Å. NHS esters react specifically with the primary amines of lysine and the N-terminal of each polypeptide[5]. Structural analysis indicates that each BrlR protomer contains only six lysine residues and all these lysines are located on the HTH motif and the C-terminal multidrug-binding domain of BrlR. The long α-helix linker of BrlR which mainly mediates BrlR tetramerization contains no lysine (Supplementary Fig. 13d). Since all cross-linkings occur between primary amines, the distances and steric hindrances between each pair of primary amines will determine the reaction result.

To make things clear we first check the contribution by the lysine residues. We measured the cross-distance matrix between each pair of primary amines of lysine from protomer A to B,C and D in apo BrlR (Supplementary Table 1 for review). The shortest distance between protomer A and protomer B is 31.4 Å (the counterpart is 35.5 Å in the BrlR-c-di-GMP structure), the shortest distance between protomer A and protomer C 31.6 Å (the counterpart is 45.0 Å in the BrlR-c-di-GMP structure), and the shortest distance between protomer A and protomer D 20.1 Å (the counterpart is 18.3 Å in the BrlR-c-di-GMP structure) (Supplementary Table 1 for review). Considering the spacer length of DSP is 12.0 Å and the length of the side chain of lysine is 6.3 Å, the intersubunit cross-linking most likely happen between protomer A and protomer D rather than between A and B or A and C. Strikingly, c-di-GMP binding may further promote the cross-linking between A and D (Supplementary Fig. 13f). For the same reason, cross-linking can also happen between protomer B and protomer C rather than B and D (Supplementary Table 3). Therefore, BrlR dimer is the major product of DSP cross-linking if lysine is the only residue gets involved in cross-linking. We thus check if the N-terminal amine also gets involved in cross-linking. Structural analysis shows that for each N-terminal amine there are two lysine residues within 20 Å (K208B and K241B is close to N-terminal amine of A, K208A and K241A close to N-terminal amine of B, K208D and K241D close to C, K208C and K241C close to D). It looks like that the N-terminal amine could be cross-linked to these two lysine residues and if this happens the major product should be tetramer considering the contribution of other lysine residues. However, two factors negatively affect the contribution of the N-terminal amine. First, Y270 makes a steric hindrance which is of no advantage to the cross-linking reaction. Secondly, the distance between K208 and K241 which belong to the same peptide chain is less than 10 Å. There is no steric hindrance between these two lysine residues. This means the cross-linking reaction between these two residues will outcompete the reaction with the N-terminal amine. Thus, only a little bit tetramers form after cross-linking.

To further clarify the contribution of the N-terminal amine we performed the cross-linking of the N-terminal His_6-taged BrlR (Its N-terminal amine would be too far to be linked to K208 or K241). The result shows that the amount of dimer almost remains the same as that of untagged BrlR while the already small amount of tetramer is further reduced (Supplementary Fig. 13e). In summary, cross-linking primarily happens between K215A and K69D (or K215C and K69B) thus produces the major product dimer. The N-terminal amine may have a little chance to be linked to K208 or K241and produces a little tetramer (Tetramer may also form by random collision between two dimers).

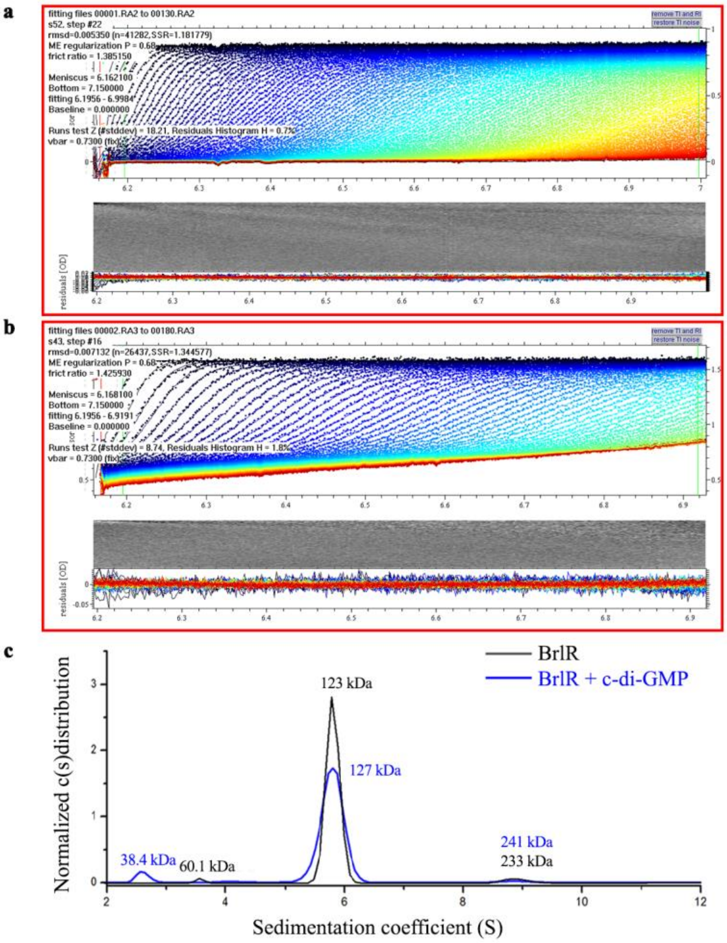

Supplementary Figure 1: The oligomer state of BrlR and BrlR with ligands. (**a** and **b**) Sedimentation coefficient distribution analysis of BrlR (**a**) and BrlR with c-di-GMP (**b**). The upper panels show the fringes collected by the interference optics, and the raw sedimentation velocity scans were overlaid with the best-fit curves obtained from sedimentation coefficient distribution analysis. The lower panels show the residual fringes after fitting the raw data with modeled curves. (**c**) Sedimentation coefficient distribution profiles of BrlR and BrlR with c-di-GMP. The calculated molecular weight of each peak is noted in the figure.

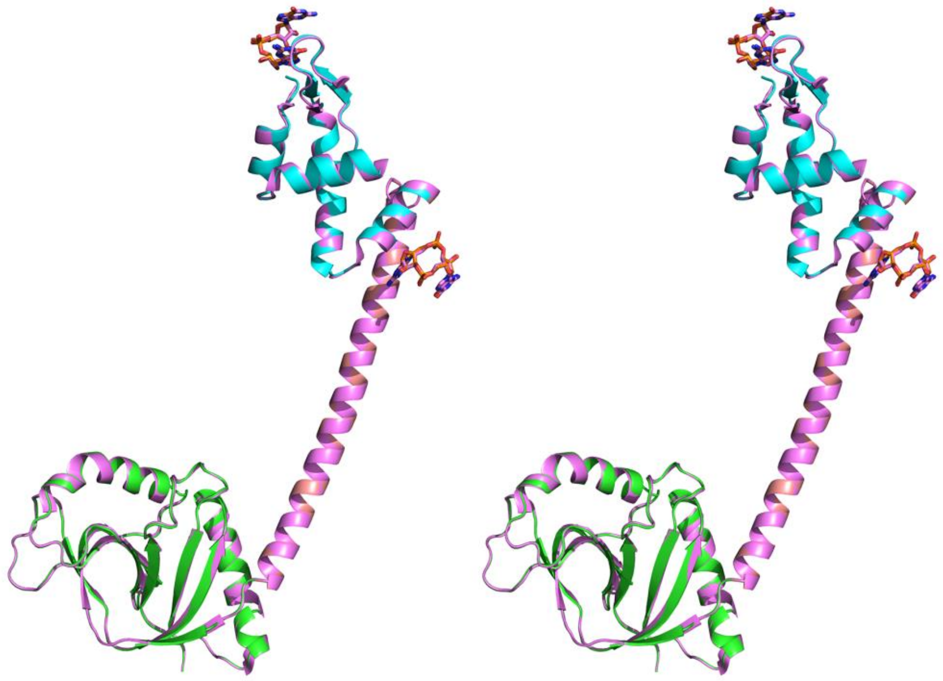

Supplementary Figure 2: Structural superpositions of our BrlR monomer and the reported BrlR-c-di-GMP complex structure (PDB code: 5XQI, color in purple) (refer) in the stereo view.

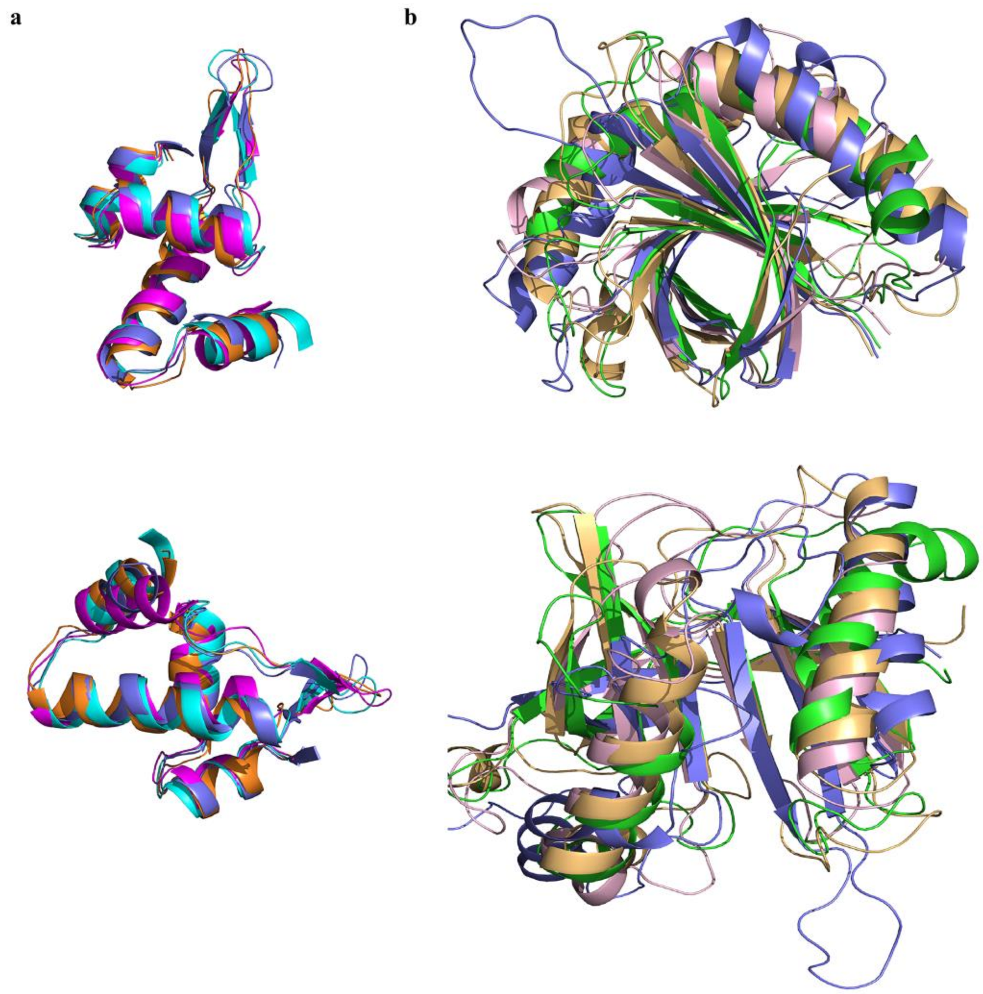

Supplementary Figure 3: Structural comparison of DNA-binding domains and multidrug-binding domains of members of MerR family. (**a**) BrlR, SoxR (PDB code: 2ZHH, colored in orange), BmrR (PDB code: 1R8E, colored in blue), and CueR (PDB code: 1Q07, colored in magenta) were compared for the DNA-binding domain. (**b**) BrlR-C, SAV2435 (PDB code: 5KAU, colored in light orange), BmrR (PDB code: 1R8E, colored in blue), and VCH_CASS2 (PDB code: 3GK6, colored in light pink) were compared for the multidrug-binding domain.

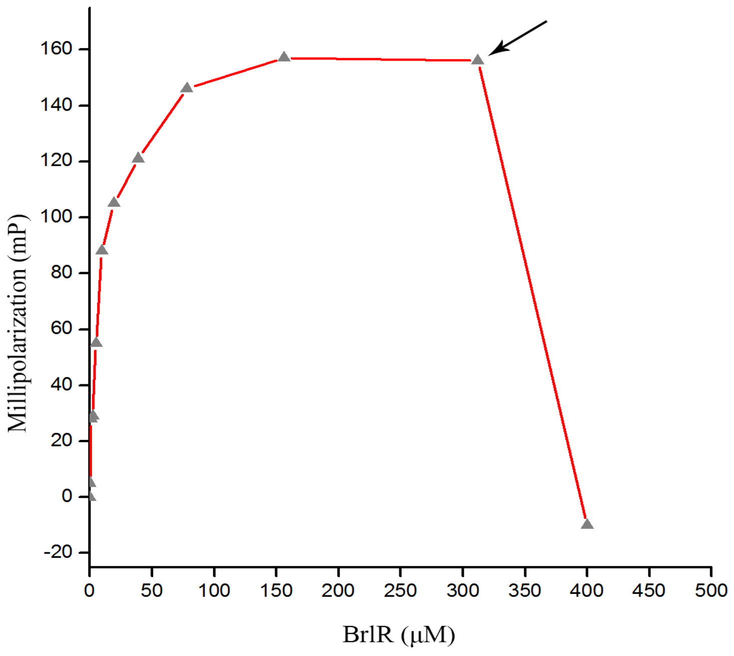

Supplementary Figure 4: Fluorescence polarization competition experiment. Wt BrlR was added to a final concentration of 320 μM in the reaction buffer containing 1 nM F-c-di-GMP, and the binding curve was recorded. The K_D value for binding was determined as 7.33 ± 0.53 μM. After the addition of 760 μM c-di-GMP (indicated with an arrow in the figure), an apparent competition with the fluoresceinated c-di-GMP could be observed by the remarkable decrease in millipolarization units.

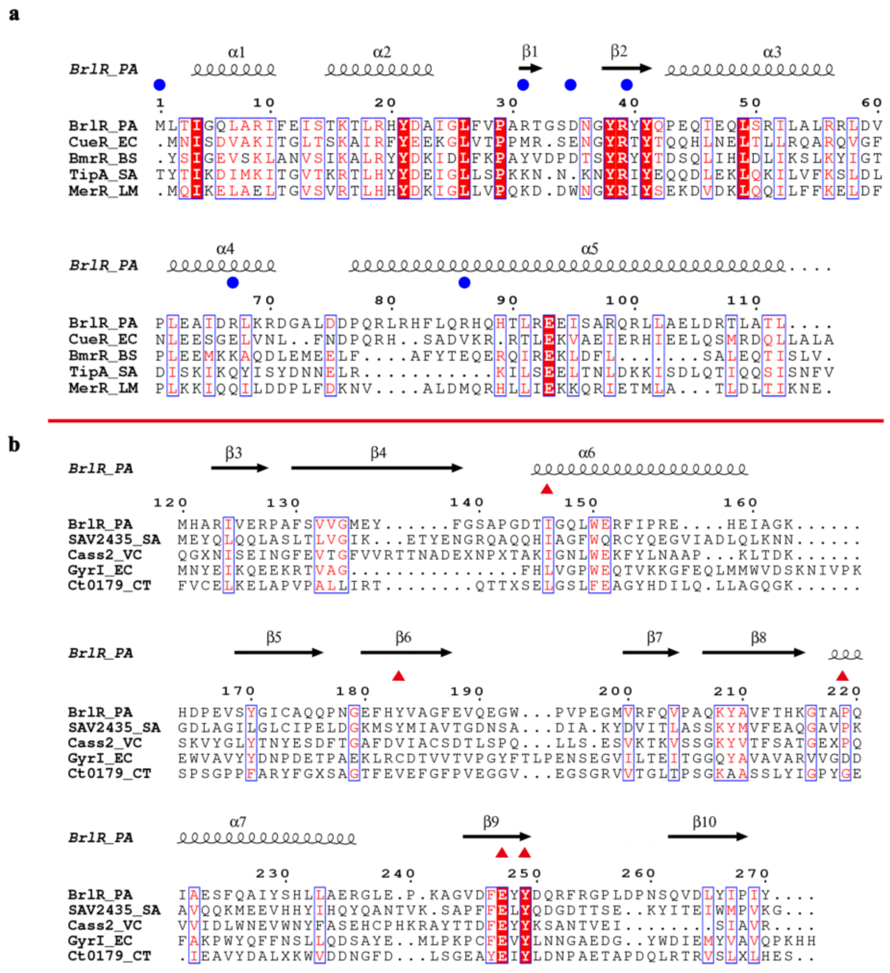

Supplementary Figure 5: Multiple sequence alignment for two terminal domains of BrlR and members in MerR family. (**a**) The DNA-binding domains of BrlR, CueR from *Escherichia coli*, BmrR from *Bacillus subtilis*, TipA from *Staphylococcus aureus*, and MerR from *Listeria monocytogenes* are shown in a multiple-sequence alignment using COBALT (www.ncbi.nlm.nih.gov/tools/cobalt) and ESPript3.0 (http://espript.ibcp.fr/ESPript/cgi-bin/ESPript.cgi). The residues involved in binding with c-di-GMP are indicated with blue dots. (**b**) The alignment of the multidrug-binding domains of BrlR from *P. aeruginosa*, SAV2435 from *S. aureus* (PDB code 5KAU), Cass2 from *Vibrio cholerae* (PDB code 3GK6), GyrI from *E. coli* (PDB code 1JYH), and Ct0179 from *Chlorobaculum tepidum* (PDB code 3E0H) were performed using ESPript 3.0. The residues involved in pyocyanin-binding are indicated with red triangles. The secondary structure for BrlR is shown in the alignment.

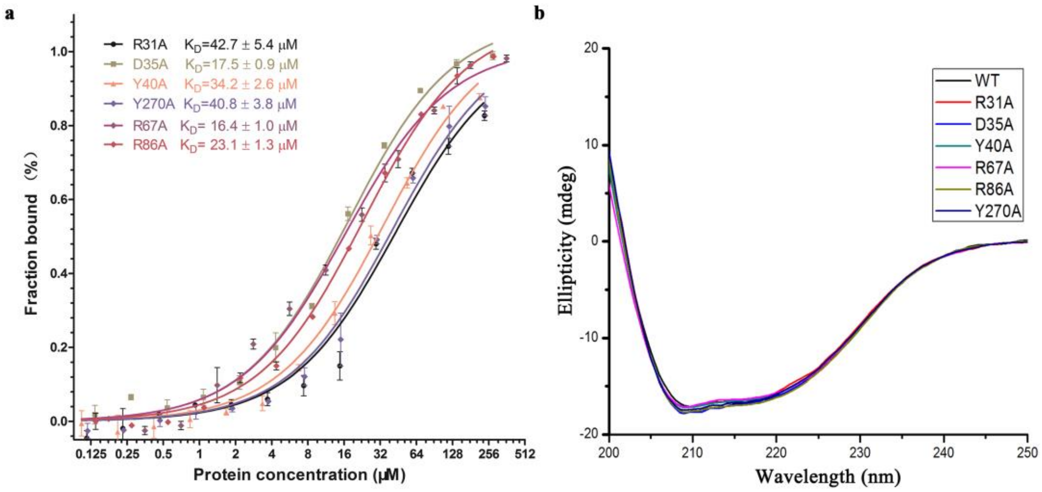

Supplementary Figure 6: FP analyses of c-di-GMP binding to related mutant BrlR proteins (**a**) Binding curves of BrlR mutants to c-di-GMP. The binding isotherms were fit to deduce the binding affinities. (b) Far-UV CD spectra (200-250 nm) of wild-type BrlR and mutants.

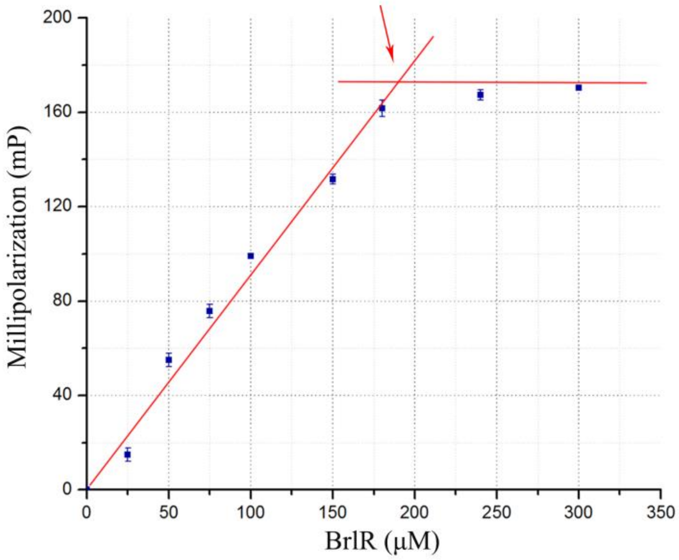

Supplementary Figure 7: Determination of the stoichiometry of BrlR binding to c-di-AMP. In the stoichiometry experiment, the inflection point occurs at a concentration of ~190 μM BrlR protein (indicated by red arrow in the figure), which suggests a shift from high-affinity binding to no binding. Thus, the binding stoichiometry of c-di-AMP to BrlR was calculated as the initial concentration of c-di-AMP (195 μM) to the BrlR concentration (190 μM) at the inflection point, revealing a 1:1 ratio of BrlR:c-di-AMP binding.

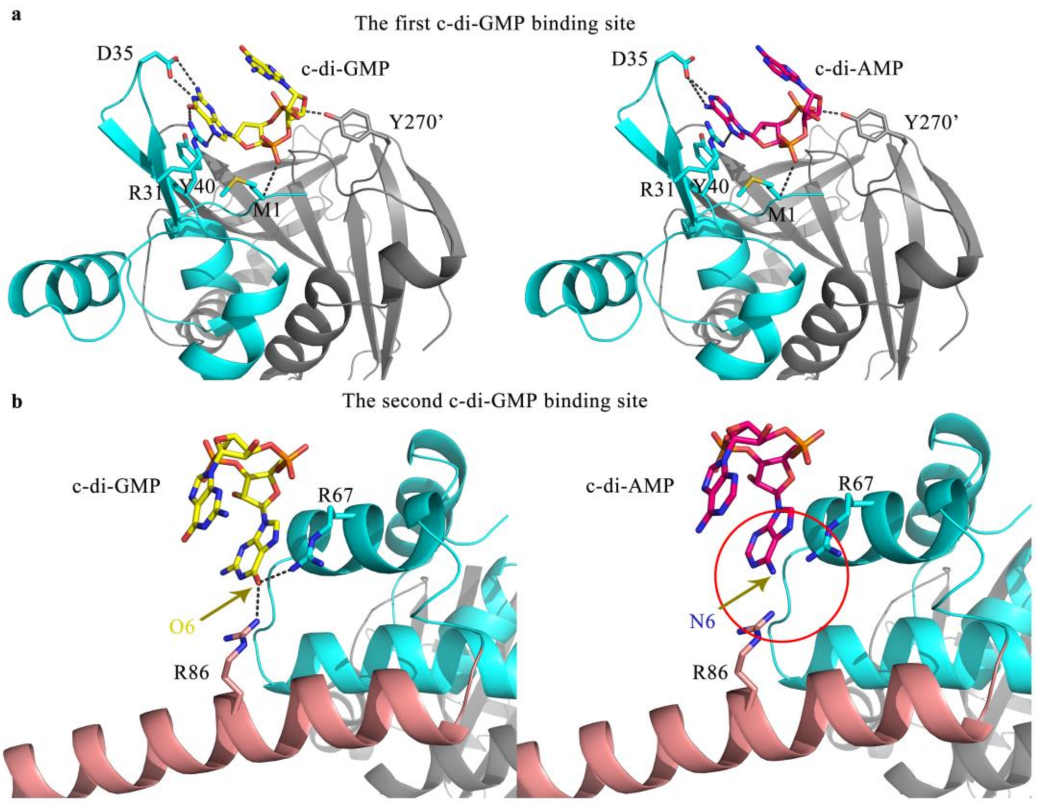

Supplementary Figure 8: Structural analysis of c-di-GMP and c-di-AMP binding to BrlR. (**a**) The bound c-di-GMP (yellow) or c-di-AMP (pink) have almost the same interactions with BrlR in the first c-di-GMP binding site. (**b**) c-di-GMP (yellow) in the second c-di-GMP binding site is stabilized by two hydrogen bonds. However, a c-di-AMP in this site will disrupt these two hydrogen bonds and produce a like charge repulsion with guanidyls of R67 and R86.

a b
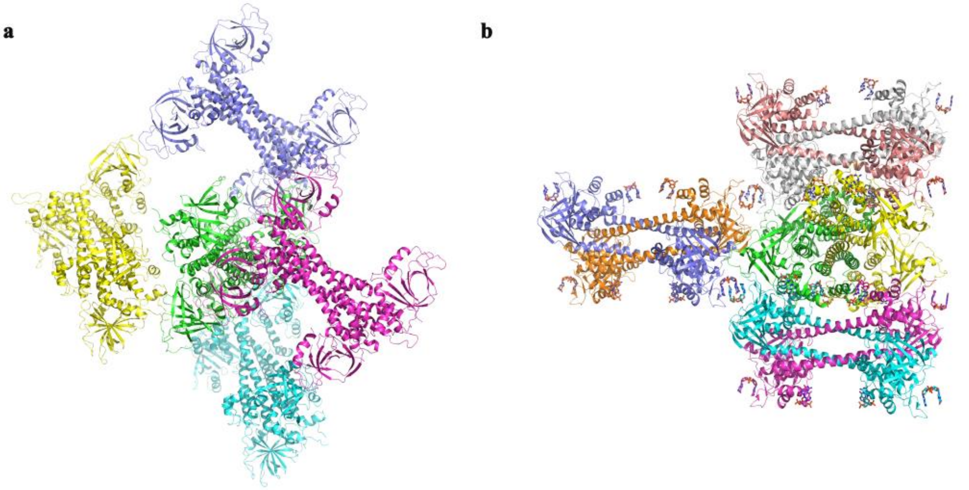

Supplementary Figure 9: Crystal packing of apo-BrlR and BrlR-c-di-GMP structures. (**a**) The apo-BrlR in the crystal lattice of the *P65* crystal is shown as cartoons. (**b**) The BrlR-c-di-GMP in the crystal lattice of the *I4* crystal is shown as cartoons. The connected c-di-GMPs between the symmetric molecules are shown as sticks.

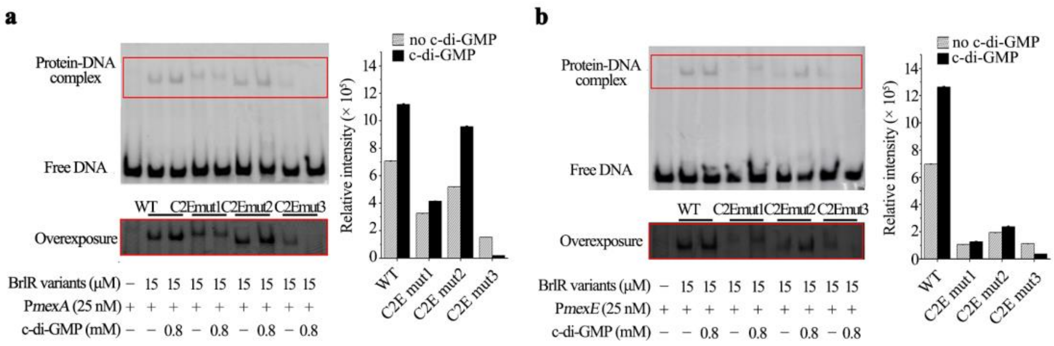

Supplementary Figure 10: EMSAs of BrlR mutants for two c-di-GMP binding sites and its target promoters. (**a**) BrlR-DNA gel mobility shift assays using BrlR mutants and P*mexA* in the absence or presence of c-di-GMP. (**b**) BrlR-DNA gel mobility shift assays using BrlR mutants and P*mexE* in the absence or presence of c-di-GMP. The protein-DNA complexes within the top red rectangle are overexposed. The results were quantified by band densitometry (right). Error bars, s.d., obtained from triplicate experiments.

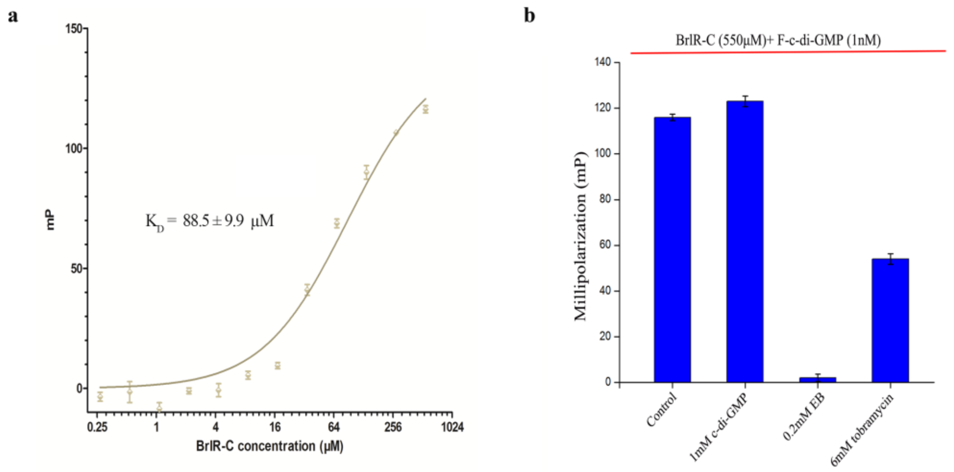

Supplementary Figure 11: FP analyses of F-c-di-GMP binding to BrlR-C. (**a**) Isotherms of BrlR-C binding to F-c-di-GMP. The binding experiment was carried out at 25 °C in a buffer containing 150 mM NaCl, 25 mM Tris·HCl (pH 7.5), and 1 nM F-c-di-GMP. Increasing concentrations of BrlR-C were titrated into the reaction mixture to obtain the binding isotherms. (**b**) Fluorescence polarization competition experiment of BrlR-C. As a control, 550 μM BrlR-C was added to the reaction tube containing 1 nM F-c-di-GMP, and different competitors were respectively added in the solution and the values of millipolarization were recorded.

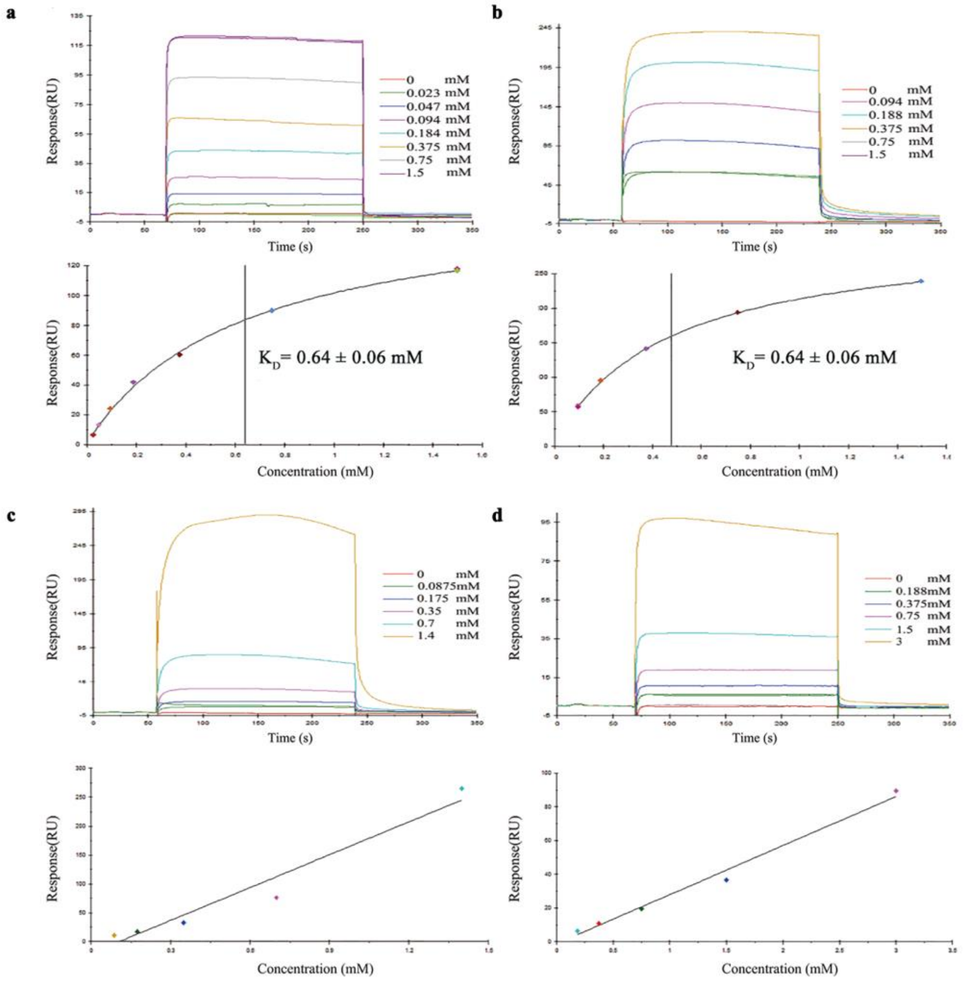

Supplementary Figure 12: SPR test for the interactions between antibiotics and BrlR. SPR sensorgrams and resulting affinity fit data for tobramycin (**a**), gentamicin (**b**), norfloxacin (**c**), and tetracycline (**d**) binding to BrlR. The concentrations of antibiotics are indicated in the figure, respectively. The antibiotic binding and dissociation phases for all sensorgrams are shown in the upper panels. The binding responses were measured 4 s before the end of the injection, and K_D values were calculated using the BIAevaluation software and shown in the lower panels.

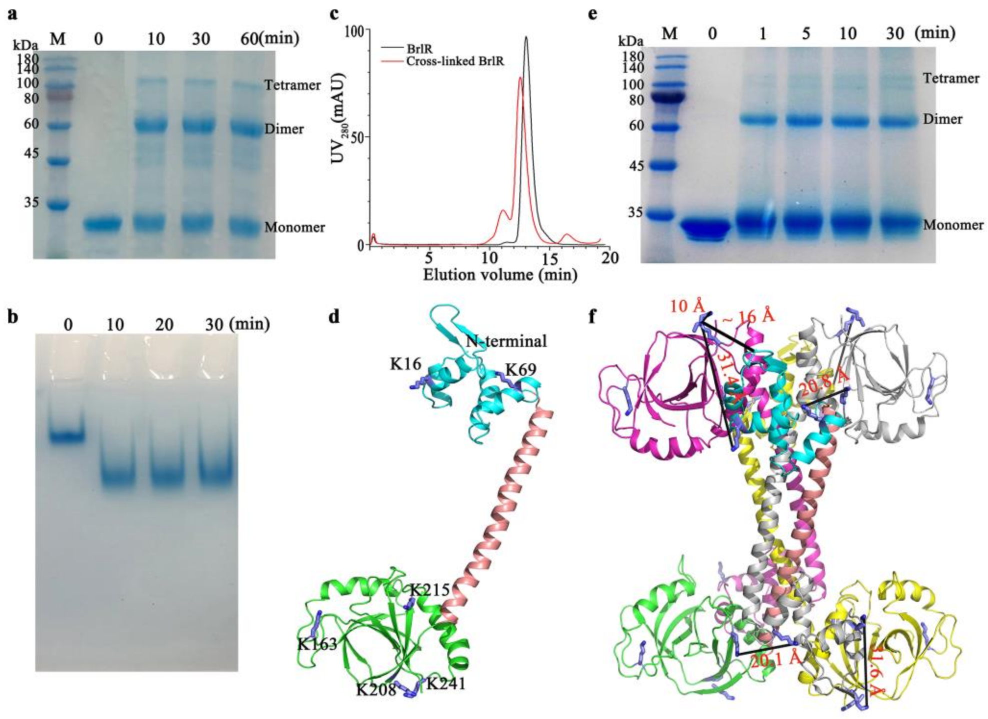

Supplementary Figure 13: BrlR is primarily cross-linked into dimers. (**a**) SDS/PAGE analyses of BrlR protein (25 μM) incubated with the primary amine-specific cross-linker DSP (1 mM) for 10, 30 and 60 min. The control protein was labeled 0 min. (**b**) The native gel analyses of the cross-linked BrlR, the wild type BrlR is a control. (**c**) Size-exclusion chromatography of BrlR and cross-linked BrlR using a Superdex 200 column. (**d**) The locations of all lysine residues in BrlR. The HTH motif contains two lysine residues and the multi-drug binding domain contains four. (**e**) SDS/PAGE analyses of the N-terminal His_6-tagged BrlR protein (25 μM) incubated with the primary amine-specific cross-linker DSP (1 mM). (**f**) The possible cross-linking between two subunits in a BrlR tetramer, the shortest distance between two lysine residues form different subunits is indicated and given in angstrom (Å).

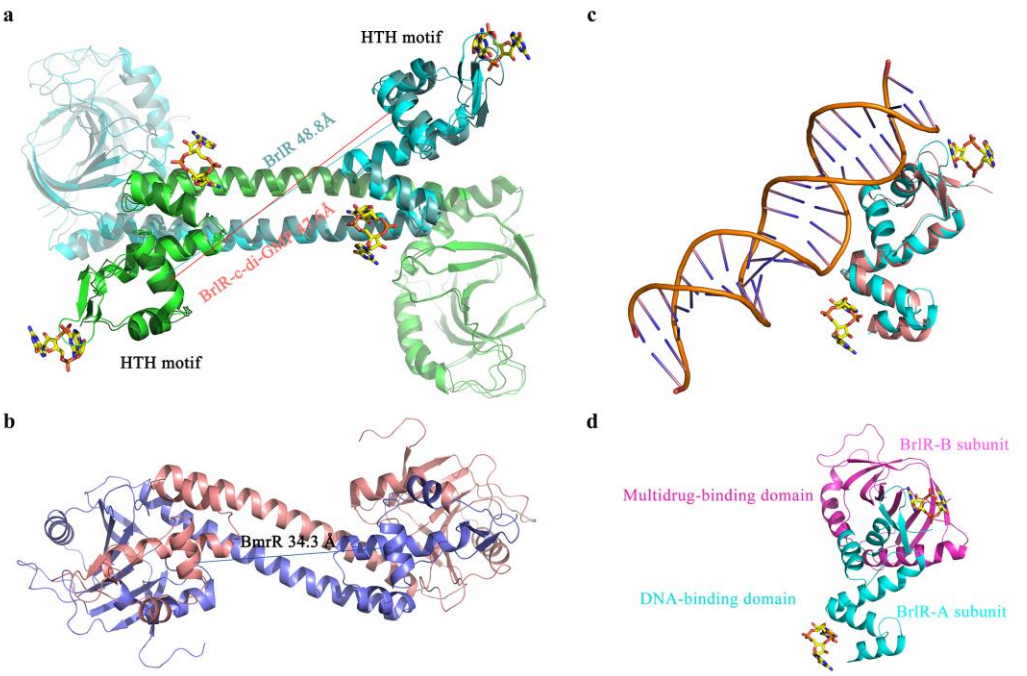

Supplementary Figure 14: The structure of BrlR differs from that of BmrR. (**a**) Superposition of the active dimer of apo BrlR (dark green and dark cyan) and c-di-GMP bound BrlR (green and cyan). The distances between recognition helices (α2(H20Cα-H20′Cα)α2′) of two HTH motifs in BrlR and BrlR-c-di-GMP are indicated and given in angstrom (Å). (**b**) The dimer structure of apo BmrR. The same distance between recognition helices (α2(Y24Cα-Y24′Cα)α2′) is indicated and given in angstrom (Å). (**c**) Superposition of the HTH motif of the BrlR-c-di-GMP (cyan) and DNA-bound BmrR(salmon). (**d**) The interface of the DNA-binding domain of subunit A and the multidrug-binding domain of subunit B with the same view as panel **c**.

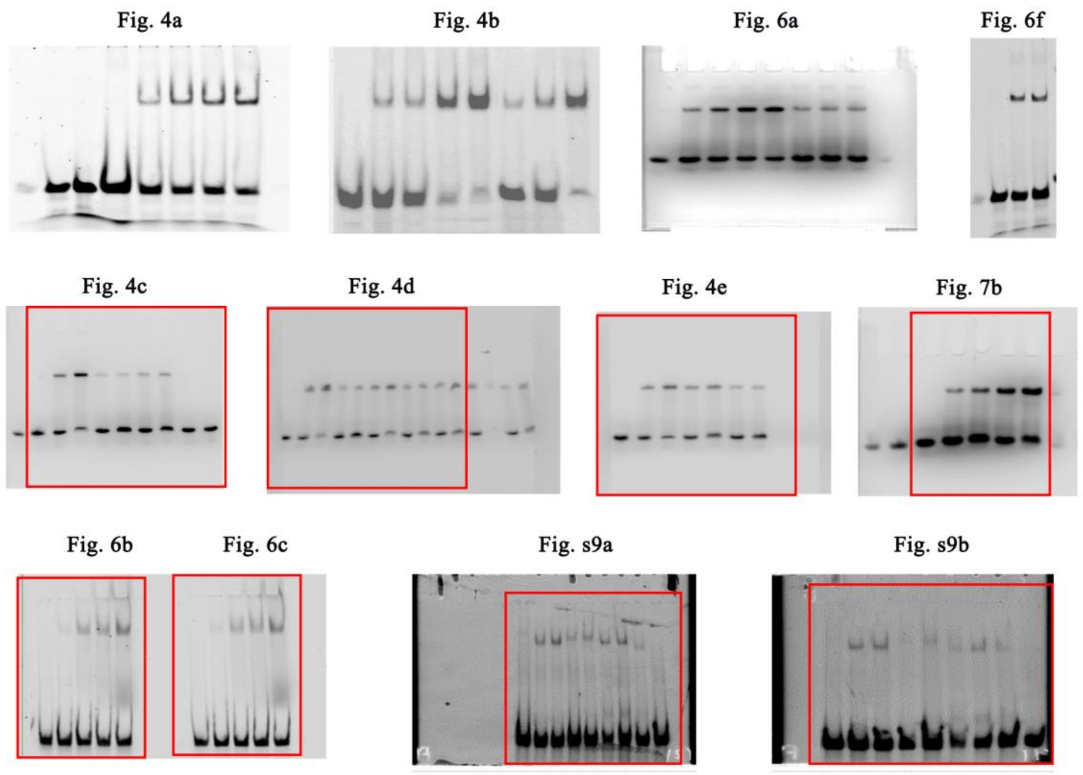

Supplementary Figure 15: The full gels of EMSAs.

Supplementary Table 1: Primers used in this study.

Primer names	Sequences (5’-3’)	Purpose
Sumo-brlR-F	CAGATTGGTGGATCCATGCTCACCATCGGCCAACTGG	molecular cloning (pET28a-sumo)
Sumo-brlR-R	GTGGTGCTCGAGCTAGTAGATGGGGATATACAGGTC	molecular cloning (pET28a-sumo)
BrlR-C-F	AGGGGCCCGGATCCATGCATGCACGAATCGTCGA	molecular cloning (pGL01)
BrlR-C-R	TTACCAGACTCGAGTCAGTAGATGGGGATATAC	molecular cloning (pGL01)
PA4843-F	ATATACATACCCATGACCGAGCACGATGACCCGACCCT G	molecular cloning (pHERD-20T)
PA4843-R	GCCAGTGCCAAGCTTTTATCGAGCGTCGGGACGATATA CC	molecular cloning (pHERD-20T)
BrlRR31A-F	ATGCCATCGGCCTGTTCGTCCCCGCGGCCACCGGTAG CG	quickchange
BrlRR31A-R	AGCGGTAGCCGTTGTCGCTACCGGTGGCCGCGGGGAC GA	quickchange
BrlRD35A-F	TGTTCGTCCCCGCGCGCACCGGTAGCGCCAACGGCTA CCG	quickchange
BrlRD35A-R	TCCGGCTGGTAGTAGCGGTAGCCGTTGGCGCTACCGG TGC	quickchange
BrlRY40A-F	CACCGGTAGCGACAACGGCTACCGCGCCTACCAGCCG GA	quickchange
BrlRY40A-R	GCTGCTCGATCTGTTCCGGCTGGTAGGCGCGGTAGCC GT	quickchange
BrlRR67A-F	TGGACGTTCCGCTGGAAGCCATCGACGCCCTGAAACG CG	quickchange
BrlRR67A-R	CGTCCAGCGCGCCATCGCGTTTCAGGGCGTCGATGGC TT	quickchange
BrlRR86A-F	CGCAACGCCTGCGGCATTTCCTGCAAGCACACCAGCA CA	quickchange
BrlRR86A-R	ATCTCTTCGCGCAGGGTGTGCTGGTGTGCTTGCAGGA AAT	quickchange
BrlRW150A-F	CCGGCGACACCATCGGCCAGCTCGCGGAACGCTTCA	quickchange
BrlRW150A-R	CGTGCTCGCGCGGGATGAAGCGTTCCGCGAGCTGGCC G	quickchange
BrlRY183A-F	CGCAGCAGCCCAACGGCGAATTCCACGCCGTCGCCGG C	quickchange
BrlRY183A-R	CTCCTGCACCTCGAAGCCGGCGACGGCGTGGAATTCG	quickchange
BrlRE247A-F	GAGCCCAAGGCGGGGGTCGACTTCGCATACTACGAC	quickchange
BrlRE247A-R	TCCGCGGAAACGCTGGTCGTAGTATGCGAAGTCGAC	quickchange
BrlRY249A-F	AGGCGGGGGTCGACTTCGAATACGCCGACCAGCGTT	quickchange
BrlRY249A-R	AGCGGTCCGCGGAAACGCTGGTCGGCGTATTCGAAGT	quickchange

BrlRY270A-F	ATTGGTGGATCCATGCTCACCATCGGCCAACTGGCGCG	quickchange
BrlRY270A-R	GTGCTCGAGTCAGGCGATGGGGATATACAGGTCGACTTG	quickchange
brlR-F	GCAACGACACCAGCACAC	RT-PCR
brlR-R	GAAGCGTTCCCAGAGCTG	RT-PCR
16S rDNA-F	GCAACGACACCAGCACAC	RT-PCR
16S rDNA-R	GAAGCGTTCCCAGAGCTG	RT-PCR
P*brlR*-F	FAM-ACCCCTTGACCTTGCCCCAGGGGCAATCCGTAGTCT	EMSA
P*brlR*-R	AGACTACGGATTGCCCCTGGGGCAAGGTCAAGGGGT	EMSA
P*mexA*-F	FAM-ACACCTATGAATGTAAGTATTTTGCCTGCCTTCTTCGAGCCGGTGCAGCGCCTTCACGCTGCATCGGCCGCTTTCGCT	EMSA
P*mexA*-R	AGCGAAAGCGGCCGATGCAGCGTGAAGGCGCTGCACCGGCTCGAAGAAGGCAGGCAAAATACTTACATTCATAGGTGT	EMSA
P*mexE*-F	FAM-AATCTCGTCGTTCGATTAGTTCCCTGCCGGAGCAGCCCGCAGACTTCGCCAATCCCGAAAAACCGACTGGCGGAGT	EMSA
P*mexE*-R	ACTCCGCCAGTCGGTTTTTCGGGATTGGCGAAGTCTGCGGGCTGCTCCGGCAGGGAACTAATCGAACGACGAGATT	EMSA

Supplementary Table 2: Bacterial strains and plasmids used in this study.

Strain or plasmid	Relevant characteristics	Source
E. coli		
DH5α	*F⁻ φ80lacZ ΔM15 Δ(lacZYA-argF)U169 recA1 endA1 hsdR17(rk⁻, mk⁺)phoA supE44 thi-1 gyrA96 relA1 tonA*	Stratagene
BL21 star (DE3)	F^- *ompT* *hsdS*$_B$ (rB^- m$_B^-$) *gal dcm met* (DE3)	Invitrogen
P. aeruginosa		
PAO1	Wild type	Our lab
Δ*brlR*	*brlR* deletion mutant of PAO1;	Jia Yin, Shandong University, China
Δ*phzA$_1$-G$_1$*/Δ*phzA$_2$-G$_2$*	No phenazine production PAO1 strain	Qing Wei, University of the Chinese Academy of Sciences, China
Δ*phzMSH*	No pyocyanin production PAO1 strain	Qing Wei, University of the Chinese Academy of Sciences, China
**Plasmids**		
pHERD-20T	*E. coli-P. aeruginosa* shuttle plasmid containing inducible P_{BAD} promoter, Ap^r	Our lab
pET28a-sumo	T7 *lac* promoter-operator, N-terminal His tag followed a sumo tag, Kan^r	Our lab
pGL01	T7 *lac* promoter-operator, N-terminal His tag, Ap^r	Our lab
pME6522	pVS1-p15A shuttle vector for transcriptional lacZ fusion and promoter probing, Tc^r	Chao Gao, Shandong University, China
pET-15b	Expression vector, N-terminal his-tag, Ap^r	This study
pHERD-20T-PA4843	PA4843 cloned into pHERD-20T; Ap^r	This study
pET28a-sumo-*brlR*	Protein expression construct, the entire gene of BrlR cloned in pET28a vector fused an N-terminal sumo tag.	This study
pET28a-sumo-*brlR C2Emut1*	pET28a-*brlR* with mutated M1, R31, D35, and Y40 to A	This study
pET28a-sumo-*brlR C2Emut2*	pET28a-*brlR* with mutated R67 and R86 to A	This study
pET28a-sumo-*brlR C2Emut3*	pET28a-*brlR* with mutated M1, R31, D35, Y40, R67, and R86 to A	This study
pGL01-*brlR*-C	Protein expression construct, BrlR-C terminal domain (120-end) cloned in pGL01 vector.	This study
pME6522-P*brlR*-BrlR	pME6522 with 300 bp upstream and coding box of *brlR* region, Tc^r	This study
pME6522-P*brlR*-BrlR	pME6522-P*brlR*-BrlR with mutated M1,	This study

C2Emut1	R31, D35, and Y40 to A in *brlR* coding box.	
pME6522-P*brlR*-BrlR C2Emut2	pME6522-P*brlR*-BrlR with mutated R67 and R86 to A in *brlR* coding box.	This study
pME6522-P*brlR*-BrlR C2Emut3	pME6522-P*brlR*-BrlR with mutated M1, R31, D35, Y40, R67, and R86 to A in *brlR* coding box.	This study

Supplementary References

1. Moreno, A. *et al.* Solution Binding and Structural Analyses Reveal Potential Multidrug Resistance Functions for SAV2435 and CTR107 and Other GyrI-like Proteins. *Biochemistry-Us* **55**, 4850-4863, doi:10.1021/acs.biochem.6b00651 (2016).
2. Bachas, S., Eginton, C., Gunio, D. & Wade, H. Structural contributions to multidrug recognition in the multidrug resistance (MDR) gene regulator, BmrR. *Proc Natl Acad Sci U S A* **108**, 11046-11051, doi:10.1073/pnas.1104850108 (2011).
3. Newberry, K. J. *et al.* Structures of BmrR-drug complexes reveal a rigid multidrug binding pocket and transcription activation through tyrosine expulsion. *J Biol Chem* **283**, 26795-26804, doi:10.1074/jbc.M804191200 (2008).
4. Holm, L. & Rosenstrom, P. Dali server: conservation mapping in 3D. *Nucleic Acids Res* **38**, W545-549, doi:10.1093/nar/gkq366 (2010).
5. Mattson, G. *et al.* A practical approach to crosslinking. *Mol Biol Rep* **17**, 167-183 (1993).
